# Effects of nurses' schedule characteristics on fatigue: An integrative review

**DOI:** 10.1097/01.NUMA.0000921904.11222.11

**Published:** 2023-03-30

**Authors:** Melita Peršolja

**Affiliations:** **Melita Peršolja** is an associate professor, Vipava Unit of Faculty of Health Sciences at the University of Primorska, Slovenia.

## Abstract

An integrative review of 21 studies found mixed results on the associations of work schedule and nurse fatigue. Suggestions for nurse leaders include monitoring fatigue in shift workers, ensuring supportive work design, promoting a healthy lifestyle, and implementing scheduling interventions.

**Figure:**
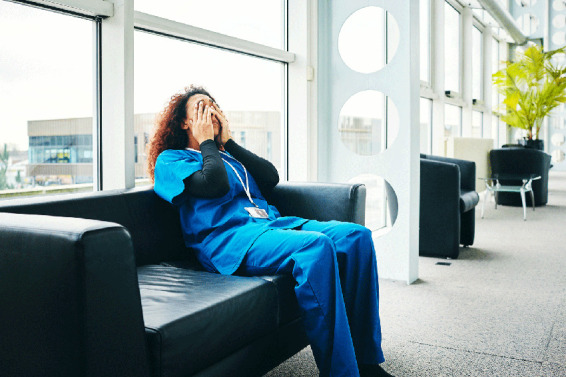
No caption available.

The organization of work in healthcare settings affects employees' health, and shift work is a strong risk factor for poor health.^1^-^3^ In nursing, the traditional schedule (7 a.m. to 3 p.m., working only during the week) is an exception. Most nurses work in shifts (hours of work/day, with staff available to work morning, evening, or night).^4^ Shift work can disturb employees' circadian rhythms and trigger a number of adverse psychological and physical changes, impacting neuro-behavioral and physiologic functioning, psychomotor performance, and menstrual regularity; interfering with sleep quality and duration; increasing the likelihood of burnout and dissatisfaction at work, fatigue, emotional exhaustion, depersonalization, decreased self-esteem, cardiovascular disease-related mortality, and the risk of developing type 2 diabetes.^5^-^14^

Shift scheduling in nursing can be roughly described as having cyclic or noncyclic patterns. In cyclic scheduling, fixed patterns of days on and off are established, and the staff is rotated continuously through them.^15^ At the other extreme, nurses are asked to sign up for those shifts that they wish to work over the planning period. Each planning period is considered independently, and new schedules are prepared at the beginning of each month. The work assignments are arranged for up to 6 weeks at a time, and each department or unit generates its own schedule.^16^ Adjustments and decisions need to be made daily, and there are many shift changes on short notice, as the nurse manager tries to resolve conflicts through consensus.^15^,^17^ Schedules are constantly changing and the manager's role is demanding because the schedules also have to comply with government regulations. The objective is to find balance between satisfying individual preferences and minimizing personnel costs.^16^,^18^,^19^

Workplace fatigue is recognized as a physiologic state of impaired performance, a multidimensional consequence of four main factors: sleep loss, extended time awake, working and sleeping at suboptimal times in the circadian body clock cycle, and excessive physical and mental workload.^20^ Fatigue is an individual experience that's defined through five dimensions: lack of energy, muscle weakness, physical discomfort, decreased motivation, and insomnia.^21^ There are also other indicators such as lack of power, slowness, and lethargy. Nurses with acute and chronic fatigue report poorer physical performance, are less alert, and are less able to concentrate and communicate effectively when providing patient care.^22^ Fatigue accumulates over time and negatively affects work performance, increasing the risk of work-related injuries, musculoskeletal disorders, stress-related illnesses, and dissatisfaction.^23^

Researchers have investigated the impacts of particular features of nurses' shift work to a limited extent. Smith-Miller identified some work characteristics (recuperation, shift work, rotating shift, 12 hours versus 8 hours, work environment, unit culture) affecting nurse fatigue.^14^ Min also analyzed different characteristics of the work schedule (number of working hours per week, normal number of working hours per shift, overtime in the previous month, number of afternoon and night shifts per month, number of quick returns between shifts, day off work) and their impact on nurses' fatigue.^24^ They found that the research results were inconsistent and the fatigue construct in each study was designed differently, and they concluded that there was insufficient evidence to confirm the association between shift-work characteristics and fatigue in nurses.

To develop strategies to effectively manage work-related fatigue in nurses, it's important to analyze scheduling in relation to fatigue.

## Methods

### Aim

The goal of the research is to examine the scientific literature to answer the question: What's the evidence that schedule characteristics are associated with work-related fatigue among hospital nursing staff working in shifts? Addressing this question will provide evidence that may help nurse leaders better understand the importance of managing the risks associated with shift work.

### Design

Using a four-step search strategy, researchers conducted an integrative review of the literature (see Table 1) and reported the results in accordance with the Whittemore and Knafl guidelines.^25^ In the initial search, investigators used Medical Subject Headings (MeSH) and the Cumulative Index to Nursing and Allied Health Literature (CINAHL) to identify suitable keywords and index terms and ensure no similar systematic review had been conducted in the last 3 years. A search strategy was developed for each database using Boolean operators (AND, OR, NOT) based on three key concepts: “fatigue,” “nurses,” and “scheduling.”

**Table 1:** Search methods

Studies were included if:	they were published in peer-reviewed journals, had shift nurses as the study population, operationalized nurse fatigue as the dependent variable and scheduling patterns as an independent variable, investigated fatigue in employed nurses (LPNs and above), had mixed samples of healthcare workers, and were written in English
Studies were excluded if:	they were conference abstracts, magazine articles, newspaper articles, commentaries, or letters to the editor, or had inaccessible full texts
The search terms were:	shift work, work schedule, nurse scheduling, standard shiftwork index, shift systems design, irregular schedules, rotating shifts, shift-work schedules, rotation rosters, fatigue, managing shift work, speed of rotation, timing of shifts, nurses

Six electronic databases were used in the searches: PubMed, Cochrane Central Register of Controlled Trials (CENTRAL), Scopus, CINAHL, ProQuest Theses and Dissertations, and Web of Science. The time frame of the searches was up to August 2021. Lastly, the reference lists of relevant studies were manually screened to identify additional relevant studies.

The search results were imported and managed using EndNote X9 (Thomson Reuters, New York, US). Duplicates were removed electronically and then manually. The full texts of potential studies were retrieved and reviewed. The primary reviewer contacted study authors to obtain the full paper via ResearchGate or email if it wasn't available in the library. A second reviewer was also used throughout the selection process.

## Results

### Search outcome

Investigators found 6,450 articles in the databases using the search method described above and evaluated the literature against fatigue and scheduling patterns. After removing duplicates and then examining the title, abstract content, and keywords, investigators chose 159 articles. The articles were then reviewed for quality using the CASP (Critical Appraisal Skills Programme) tool.^26^

**Figure 1:**
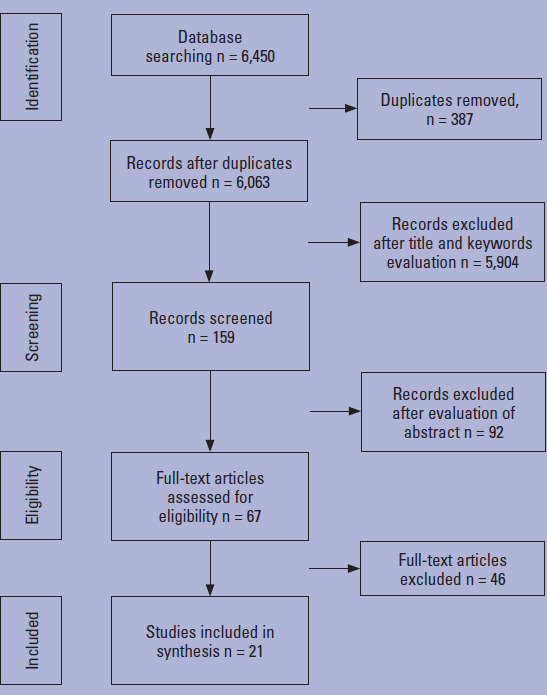
PRISMA diagram of article selection

The PRISMA method (Preferred Reporting Items for Systematic Reviews and Meta-Analyses) was used to report the article selection process (see Figure 1).^27^ Twenty-one articles were selected for final analyses (see Table 2). Most of them (n = 13) were cross-sectional studies, six were literature reviews, and two were case-control studies.

**Table 2:** Characteristics of included studies

Author, year	Country	Design	Sample, setting	Measurement of fatigue
Afrasiabifar et al., 2018	Iran	Case-control	46 nurses	Multidimensional Fatigue Inventory
Barker and Nussbaum, 2011	US	Cross-sectional	745 nurses	Fatigue in Nursing Survey Set
Dall'Ora et al., 2016	UK	Literature review	35 studies	-
Driscoll et al., 2007	Australia	Literature review	44 studies	-
Ferri et al., 2016	Italy	Cross-sectional	232 nurses	Standard Shiftwork Index
Flo et al., 2014	Norway	Cross-sectional	1,224 nurses	Chalder fatigue scale
Gander et al., 2019	New Zealand	Cross-sectional	3,133 nurses	Sleep problem, sleepiness, recalling a fatigue-related error, feeling close to falling asleep at the wheel
Garrubba and Joseph, 2019	Australia	Literature review	15 studies	-
Gifkins et al., 2020	Australia	Literature review	31 studies	-
Geiger-Brown et al., 2012	US	Case-control study	80 nurses	Occupational Fatigue Exhaustion Recovery (OFER) scale
Han et al., 2014	South Korea	Cross-sectional	80 nurses	OFER scale
Hazzard et al., 2013	US	Cross-sectional	20 nurses	OFER scale
Jones et al., 2015	France	Cross-sectional	682 healthcare workers	Nottingham Health Profile, energy level, current fatigue state
Jung and Lee, 2015	South Korea	Cross-sectional	660 nurses	Insomnia, fatigue, and depression
Juniartha et al., 2020	Indonesia	Cross-sectional	45 nurses	OFER and Expanded Nursing Stress Scale questionnaires
McElroy et al., 2020	US	Cross-sectional	1,563 hospital employees	Chronic Fatigue Scale
Min et al., 2019	South Korea	Literature review	8 studies	-
Ruggiero, 2003	US	Cross-sectional	142 nurses	Standard Shiftwork Index, Chronic Fatigue Scale
Querstret et al., 2020	UK	Literature review	32 studies	-
Sagherian et al., 2017	US	Cross-sectional	77 nurses	OFER scale
Yuan et al., 2011	Taiwan	Cross-sectional	107 nurses	Questionnaire by the Japanese Association for Industrial Health

### Data abstraction and synthesis

With the open coding system used for thematic analysis, investigators identified 53 codes, which were combined into four themes: fatigue symptoms, when/how schedule characteristics, schedule-related causes of fatigue, and organizational fatigue facilitators (see Table 3).

**Table 3:** Codes and themes related to schedule patterns and fatigue

Themes	Codes	References
Fatigue symptoms	Feelings of fatigue, shift-work disorders, pathologic fatigue, intershift fatigue, acute fatigue, tired current state, daily fatigue, work-related fatigue, fatigue, drowsiness, lack of energy, difficulty concentrating, feeling uncomfortable	24,28,31-36,41
When/how schedule characteristics	Rotating night shift, rotating shift, shift rotations, night shifts, quick returns, roster changes, overtime, extensions, longer shifts, length of shift, night shifts, rotating direction, speed of rotation, differing work schedules, work arrangements, roster patterns, shift patterns, poor scheduling, shift-work schemes, work schedule characteristics	24,28,29,31-33,36,41,42
Schedule-related causes of fatigue	Disruption of circiadian rhythm, sleep deprivation, insufficient recovery time, high work/job demands, greater patient/nurse ratio, night shift	31,34,40,41
Organizational fatigue recovery facilitators	Social support, control over shift scheduling, work control, breaks, exercise intervention, choice over shift-work schedules, shorter working hours, workplace autonomy, high job control, 8-hour shifts, rotating shifts, shift-work scheme	32,39,41,46,52

### Findings

In the analyzed studies, the construct of fatigue was described with feelings of fatigue, fatigue-related clinical errors, intershift, acute or chronic fatigue, drowsiness, lack of energy, difficulties in concentrating, and feeling uncomfortable.^28^-^36^

Work schedules that include overtime, more workdays, and night shifts are said to be a significant predictor of chronic fatigue in nurses.^37^,^38^ The literature also describes the following schedule characteristics as important: rapid changes in schedule, the direction and speed of shift rotation, shift length (mainly 12-hour shifts), shift start and finish times, short breaks between shifts and quick returns, work on scheduled days off, and a high number of hours per week (over 60 hours) (see Table 4).^28^,^29^,^32^-^34^,^37^,^39^-^43^

**Table 4:** Results of included studies

Author, Year	Results
Afrasiabifar et al., 2018	A statistically significant difference was observed in mean scores of total fatigue and physical and mental subscales between the two groups after core stability exercises.
Barker and Nussbaum, 2011	Longer shift lengths and hours worked per week were associated with increases in physical and total fatigue levels. Mental, physical, and total fatigue levels also differed with shift schedule.
Dall'Ora et al., 2016	Shifts of 12 hours or longer are associated with jeopardized outcomes. Working more than 40 hours per week is associated with adverse events. Working rotating shifts was associated with worse job performance outcomes, whereas fixed night shifts appeared to enable resynchronization.
Driscoll et al., 2007	Two main aspects of shift design were considered—the direction of shift rotation and extended shift length (mainly 12-hour shifts). There's insufficient evidence to support definitive conclusions.
Ferri et al., 2016	No significant differences between rotating night-shift and day-shift groups in general feelings of fatigue.
Flo et al., 2014	Quick returns increased the risk of shift-work disorders and pathologic fatigue over a 1 year follow-up period.
Gander et al., 2019	The risk of sleep problems increased with roster changes, night shifts, and shift extensions greater than 30 minutes and decreased with more choices about shifts. Recalling a fatigue-related error was lower in more experienced nurses.
Garrubba and Joseph, 2019	The two major causes of fatigue are disruption of circadian rhythm sleep and sleep deprivation. Fatigue can be caused by work-related factors, including roster patterns, length of shift, poor work scheduling and planning the timing of shifts, insufficient recovery time between shifts, and more.
Gifkins et al., 2020	Recovery from fatigue is most significantly affected by high work and job demands, when and how of shift-work schedules. Rotating shifts were associated with higher levels of fatigue in shift-working nurses when compared with day-only nurses.
Geiger-Brown et al., 2012	Fatigue was high in one-third of nurses, with intershift fatigue (not feeling recovered from the previous shift at the start of the next shift) being most prominent.
Han et al., 2014	Shift rotations were significantly associated with acute fatigue. In comparison, when work schedules are fixed on either day or night, nurses can adapt to their schedules and may find successful strategies to reduce fatigue and increase recovery.
Hazzard et al., 2013	Despite high acute fatigue scores, intershift fatigue scores reflected recovery and chronic fatigue scores were low.
Jones et al., 2015	Female gender was associated with higher stress levels and greater fatigue. Greater social support from supervisors or colleagues decreased stress and fatigue. Longer shifts were associated with a tired current state; personnel on rotating shifts had lower stress and better current state, whereas those on night shifts had greater sleep and energy difficulties.
Jung and Lee, 2015	All variables except for three (number of children, body mass index, and working hours) were related to at least one of the symptoms associated with shift-work tolerance.
Juniartha et al., 2020	There was a significant difference in the effects of the shift-work schemes on nurses' work-related fatigue. There was a significant difference in the influence of the work distribution system on work-related fatigue.
McElroy et al., 2020	Findings suggest that night and long shifts can have negative effects on fatigue, family, and social life.
Min et al., 2019	The reviewed studies provided mixed results regarding the associations between work schedule characteristics and nurse fatigue.
Ruggiero, 2003	Variables contributing to chronic fatigue are global sleep quality and depression. Day and night nurses had similar levels of chronic fatigue.
Querstret et al., 2020	Longer shifts, shift patterns including nights, increased sleepiness, and levels of fatigue. The literature related to sleep-related/fatigue-management interventions for nurses and midwives is fragmented and lacks cohesion.
Sagherian et al., 2017	Work schedules that include overtime and more workdays can result in fatigue.
Yuan et al., 2011	Differing work schedules result in differing levels of fatigue, with shift work attributed to higher levels than day work. Nurses who worked in shifts were more fatigued than nurses who worked during the day.

Moreover, some authors have addressed schedule patterns per se as a factor connected with fatigue. Garrubba and Joseph, Yuan et al., Gifkins et al., Han et al., and Gander et al. found that rotating shifts are associated with higher levels of fatigue, especially shift patterns that include nights.^31^-^33^,^36^,^39^,^40^ When and how shift work takes place can cause both acute and chronic fatigue, but according to some authors has no significant effect on the health of nurses or the feelings of fatigue.^24^,^28^,^31^,^44^

Managers can facilitate employees' recovery from fatigue by simultaneously implementing healthy lifestyle promotion (with the focus on sleep promotion) and supportive work design—ensuring social support, greater work control, regular breaks, appropriate work and job demands, workplace autonomy, and high job control.^39^,^41^,^45^,^46^ It's recommended to provide special monitoring of shift workers to recognize fatigue symptoms early. Managers should also control shift scheduling and implement scheduling interventions, such as avoiding overtime work and long work shifts, minimizing the number of night shifts and shift rotation, using forward rotation instead of backward rotation, and ensuring enough rest time between shifts (see Table 5).^24^,^31^-^33^,^35^-^41^,^43^,^44^,^47^

**Table 5:** Recommendations for managers

Recommendation	References
Ensure supportive work design	39,41,45,46
Monitor fatigue in shift workers	24,28,29,37,41
Promote healthy lifestyle	34,36,40,50,52
Implement scheduling interventions	24,31-33,36,38,44,48

## Discussion

Our findings show that fatigue is common in nurses, but the evidence about its association with scheduling isn't consistent. Although much of the research identifies a relationship among nurse fatigue and different aspects of scheduling, there are also studies that claim shift work has no significant effect on fatigue.

Studies show the multidimensional nature of fatigue, including the variety of fatigue symptoms and measures used to assess it. Some research instruments directly measured fatigue symptoms (such as feelings of fatigue), but there were also studies with a wider research focus that included fatigue (such as the Shift Work Index).^24^,^28^,^31^-^36^,^41^ It seems that researchers can't agree on a representative instrument to measure different aspects of organization-related fatigue.

Because fatigue is subjective and the individual isn't necessarily aware of it, it's vital for the nurse manager to prevent and recognize the fatigue in employees at an early stage.^48^ Authors suggest that leaders be alert to fatigue-related clinical errors, drowsiness, lack of energy, difficulties in concentrating, and discomfort.^32^,^36^ Additionally, a standardized instrument to measure fatigue is needed.

Organizing a schedule is a challenge for nurse managers. It seems that shift work leads to fatigue regardless of the scheduling characteristics because fatigue originates from disruption of the circadian rhythm, insufficient recovery time, and high work/job demands.^39^-^41^,^49^ Managers should consider many characteristics of schedules (shift rotations, night shifts, quick returns, roster changes, overtime, length of shift, differing work schedules, work arrangements, roster patterns) that can disrupt employees' circadian rhythms.^24^,^28^,^31^-^33^,^36^,^39^-^41^

Working in shifts is almost inevitable for nurses; the real dilemma is what schedule characteristics a manager should be most concerned about when trying to minimize nurse fatigue. The law prescribes the minimum time between shifts, the minimum days off per week, and the maximum work hours per week. Other schedule characteristics are mostly in the hands of, and a challenge for, the manager. Therefore, the nurse manager's awareness can be a factor that influences the schedule properties and consequently impacts fatigue in employees.

Nurses who work shifts are exposed to a great number of adverse reactions at the same time. Shift work affects bodily functions, most notably sleep, autonomic vegetative processes, and work ability.^50^ Although difficult, it shouldn't be impossible to make a worker-friendly schedule, providing known recovery facilitators (at minimum) such as control over shift scheduling, breaks, and choice over shift-work schedules.^32^,^39^,^41^,^46^,^51^ Choice over shift-work schedules is maybe the most challenging. It could be a statement of workplace autonomy, but on the other hand, it can lead to frequent changes in schedule plans.

This review has a number of limitations. The most obvious is that shift work also includes night work, which is a known factor influencing fatigue. In some studies, shift work was used as a synonym for night work, and many studies concentrated on sleep quantity and quality. It was also difficult to compare the findings from studies because different measures were used, and researchers focused on different aspects of shift work. Although the impact of schedule characteristics on fatigue is unclear in the literature, it should be addressed in more detail in future research. We recommend case-control and longitudinal studies with standardized instruments, as well as research addressing additional variables (such as work-life balance and quality of life).

## Conclusion

The reviewed studies provided mixed results on the associations of work schedule with nurse fatigue, although in most of the literature, varied work schedules contributed to nursing fatigue. This implies that a possible association between shift work and fatigue might depend on schedule characteristics. Amid a very disrupted workforce and global nursing shortage, staffing is an issue for most, if not all, nurse managers, and they should consider schedule characteristics that minimize fatigue. Increasing nurse leaders' awareness of work design and schedule characteristics that might impose fatigue, early screening of fatigue, and promotion of a healthy lifestyle could prevent and reduce the possible negative effects of working shifts and should be integrated in nursing staff management.

## References

[1] Trybou J Germonpre S Janssens H Job-related stress and sickness absence among Belgian nurses: a prospective study. *J Nurs Scholarsh*. 2014;46(4):292–301. 24754533 10.1111/jnu.12075

[2] Baydoun M Dumit N Daouk-Öyry L. What do nurse managers say about nurses' sickness absenteeism? A new perspective. *J Nurs Manag*. 2016;24(1):97–104. 25580638 10.1111/jonm.12277

[3] Stevens RG Hansen J Costa G Considerations of circadian impact for defining ‘shift work’ in cancer studies: IARC Working Group Report. *Occup Environ Med*. 2011;68(2):154–162. 20962033 10.1136/oem.2009.053512

[4] Bae S-H Fabry D. Assessing the relationships between nurse work hours/overtime and nurse and patient outcomes: systematic literature review. *Nurs Outlook*. 2014;62(2):138–156. 24345613 10.1016/j.outlook.2013.10.009

[5] Vedaa Ø Harris A Bjorvatn B Systematic review of the relationship between quick returns in rotating shift work and health-related outcomes. *Ergonomics*. 2016;59(1):1–14. 26072668 10.1080/00140139.2015.1052020

[6] Di Muzio M Reda F Diella G Not only a problem of fatigue and sleepiness: changes in psychomotor performance in Italian nurses across 8-h rapidly rotating shifts. *J Clin Med*. 2019;8(1):47. 30621274 10.3390/jcm8010047 PMC6352064

[7] Driscoll TR Grunstein RR Rogers NL. A systematic review of the neurobehavioural and physiological effects of shiftwork systems. *Sleep Med Rev*. 2007;11(3):179–194. 17418596 10.1016/j.smrv.2006.11.001

[8] Chang W-P Chang Y-P. Meta-analysis comparing menstrual regularity and dysmenorrhea of women working rotating shifts and fixed day shifts. *J Womens Health (Larchmt)*. 2021;30(5):722–730. 32907443 10.1089/jwh.2020.8517

[9] Costa G Anelli MM Castellini G Fustinoni S Neri L. Stress and sleep in nurses employed in “3 × 8” and “2 × 12” fast rotating shift schedules. *Chronobiol Int*. 2014;31(10):1169–1178. 25216205 10.3109/07420528.2014.957309

[10] Stimpfel AW Sloane DM Aiken LH. The longer the shifts for hospital nurses, the higher the levels of burnout and patient dissatisfaction. *Health Aff (Millwood)*. 2012;31(11):2501–2509. 23129681 10.1377/hlthaff.2011.1377 PMC3608421

[11] Dall'Ora C Griffiths P Ball J Simon M Aiken LH. Association of 12 h shifts and nurses' job satisfaction, burnout and intention to leave: findings from a cross-sectional study of 12 European countries. *BMJ Open*. 2015;5(9):e008331. 10.1136/bmjopen-2015-008331 PMC4577950 26359284

[12] Gu F Han J Laden F Total and cause-specific mortality of U.S. nurses working rotating night shifts. *Am J Prev Med*. 2015;48(3):241–252. 25576495 10.1016/j.amepre.2014.10.018 PMC4339532

[13] Colditz GA Philpott SE Hankinson SE. The impact of the nurses' health study on population health: prevention, translation, and control. *Am J Public Health*. 2016;106(9):1540–1545. 27459441 10.2105/AJPH.2016.303343 PMC4981811

[14] Smith-Miller CA Shaw-Kokot J Curro B Jones CB. An integrative review: fatigue among nurses in acute care settings. *J Nurs Adm*. 2014;44(9):487–494. 25148403 10.1097/NNA.0000000000000104

[15] Bard JF Purnomo HW. Short-term nurse scheduling in response to daily fluctuations in supply and demand. *Health Care Manag Sci*. 2005;8(4):315–324. 16379414 10.1007/s10729-005-4141-9

[16] Purnomo HW Bard JF. Cyclic preference scheduling for nurses using branch and price. *Naval Research Logistics (NRL)*. 2007;54(2):200–220.

[17] Kullberg A Bergenmar M Sharp L. Changed nursing scheduling for improved safety culture and working conditions—patients' and nurses' perspectives. *J Nurs Manag*. 2016;24(4):524–532. 26762216 10.1111/jonm.12352

[18] Lin R-C Sir MY Sisikoglu E Pasupathy K Steege LM. Optimal nurse scheduling based on quantitative models of work-related fatigue. *IIE Trans Healthc Syst Eng*. 2013;3(1):23–38.

[19] Galatsch M Li J Derycke H Müller BH Hasselhorn HM. Effects of requested, forced and denied shift schedule change on work ability and health of nurses in Europe—results from the European NEXT-Study. *BMC Public Health*. 2013;13:1137. 24308567 10.1186/1471-2458-13-1137 PMC3878997

[20] ICAO. *Manual for the Oversight of Fatigue Management Approaches*. International Civil Aviation Organization; 2016.

[21] Åhsberg E. Dimensions of fatigue in different working populations. *Scand J Psychol*. 2000;41(3):231–241. 11041305 10.1111/1467-9450.00192

[22] Sagherian K Geiger Brown J. In-depth review of five fatigue measures in shift workers. *Fatigue*. 2016;4(1):24–38.

[23] Jensen HI Markvart J Holst R Shift work and quality of sleep: effect of working in designed dynamic light. *Int Arch Occup Environ Health*. 2016;89(1):49–61. 25893465 10.1007/s00420-015-1051-0 PMC4700071

[24] Min A Min H Hong HC Work schedule characteristics and fatigue among rotating shift nurses in hospital setting: an integrative review. *J Nurs Manag*. 2019;27(5):884–895. 30737987 10.1111/jonm.12756

[25] Whittemore R Knafl K. The integrative review: updated methodology. *J Adv Nurs*. 2005;52(5):546–553. 16268861 10.1111/j.1365-2648.2005.03621.x

[26] CASP. CASP Checklists. https://casp-uk.net/casp-tools-checklists. Accessed September 12, 2020.

[27] Moher D Liberati A Tetzlaff J Altman DG. Preferred reporting items for systematic reviews and meta-analyses: the PRISMA statement. *PLoS Med*. 2009;6(7):e1000097. 19621072 10.1371/journal.pmed.1000097 PMC2707599

[28] Ferri P Guadi M Marcheselli L Balduzzi S Magnani D Di Lorenzo R. The impact of shift work on the psychological and physical health of nurses in a general hospital: a comparison between rotating night shifts and day shifts. *Risk Manag Healthc Policy*. 2016;9:203–211. 27695372 10.2147/RMHP.S115326 PMC5028173

[29] Juniartha IGN Sardjono TW Ningsih DK. A comparison of work-related fatigue and stress among emergency department nurses working in 7–7-10 and 12–12 shifts at the hospitals in Badung and Denpasar. *Enferm Clin*. 2020;30(7):74–77.

[30] Min A Kim YM Yoon YS Hong HC Kang M Scott LD. Effects of work environments and occupational fatigue on care left undone in rotating shift nurses. *J Nurs Scholarsh*. 2021;53(1):126–136. 33205904 10.1111/jnu.12604

[31] Querstret D O'Brien K Skene DJ Maben J. Improving fatigue risk management in healthcare: a systematic scoping review of sleep-related/fatigue-management interventions for nurses and midwives. *Int J Nurs Stud*. 2020;106:103513. 32283414 10.1016/j.ijnurstu.2019.103513

[32] Gander P O'Keeffe K Santos-Fernandez E Huntington A Walker L Willis J. Fatigue and nurses' work patterns: an online questionnaire survey. *Int J Nurs Stud*. 2019;98:67–74. 31319337 10.1016/j.ijnurstu.2019.06.011

[33] Han K Trinkoff AM Geiger-Brown J. Factors associated with work-related fatigue and recovery in hospital nurses working 12-hour shifts. *Workplace Health Saf*. 2014;62(10):409–414. 25199168 10.3928/21650799-20140826-01

[34] Geiger-Brown J Rogers VE Trinkoff AM Kane RL Bausell RB Scharf SM. Sleep, sleepiness, fatigue, and performance of 12-hour-shift nurses. *Chronobiol Int*. 2012;29(2):211–219. 22324559 10.3109/07420528.2011.645752

[35] Jung H-S Lee B. Contributors to shift work tolerance in South Korean nurses working rotating shift. *Appl Nurs Res*. 2015;28(2):150–155. 25448057 10.1016/j.apnr.2014.09.007

[36] Yuan SC Chou MC Chen CJ Influences of shift work on fatigue among nurses. *J Nurs Manag*. 2011;19(3):339–345. 21507104 10.1111/j.1365-2834.2010.01173.x

[37] Sagherian K Clinton ME Abu-Saad Huijer H Geiger-Brown J. Fatigue, work schedules, and perceived performance in bedside care nurses. *Workplace Health Saf*. 2017;65(7):304–312. 27872407 10.1177/2165079916665398

[38] McElroy SF Olney A Hunt C Glennon C. Shift work and hospital employees: a descriptive multi-site study. *Int J Nurs Stud*. 2020;112:103746. 32928504 10.1016/j.ijnurstu.2020.103746

[39] Gifkins J Johnston A Loudoun R Troth A. Fatigue and recovery in shiftworking nurses: a scoping literature review. *Int J Nurs Stud*. 2020;112:103710. 32912638 10.1016/j.ijnurstu.2020.103710

[40] Garrubba M Joseph C. The impact of fatigue in the healthcare setting: a scoping review. Centre for Clinical Effectiveness, Monash Health. 2019. https://monashhealth.org/wp-content/uploads/2020/03/Health-worker-fatigue_Scoping-Review2019_FINAL.pdf.

[41] Jones G Hocine M Salomon J Dab W Temime L. Demographic and occupational predictors of stress and fatigue in French intensive-care registered nurses and nurses' aides: a cross-sectional study. *Int J Nurs Stud*. 2015;52(1):250–259. 25443305 10.1016/j.ijnurstu.2014.07.015

[42] Flo E Pallesen S Moen BE Waage S Bjorvatn B. Short rest periods between work shifts predict sleep and health problems in nurses at 1-year follow-up. *Occup Environ Med*. 2014;71(8):555–561. 24919881 10.1136/oemed-2013-102007

[43] Barker LM Nussbaum MA. Fatigue, performance and the work environment: a survey of registered nurses. *J Adv Nurs*. 2011;67(6):1370–1382. 21352271 10.1111/j.1365-2648.2010.05597.x

[44] Dall'Ora C Ball J Recio-Saucedo A Griffiths P. Characteristics of shift work and their impact on employee performance and wellbeing: a literature review. *Int J Nurs Stud*. 2016;57:12–27. 27045561 10.1016/j.ijnurstu.2016.01.007

[45] Hazzard B Johnson K Dordunoo D Klein T Russell B Walkowiak P. Work- and nonwork-related factors associated with PACU nurses' fatigue. *J Perianesth Nurs*. 2013;28(4):201–209. 23886284 10.1016/j.jopan.2012.06.010

[46] Fang J Qiu C Xu H You G. A model for predicting acute and chronic fatigue in Chinese nurses. *J Adv Nurs*. 2013;69(3):546–558. 22551117 10.1111/j.1365-2648.2012.06029.x

[47] Driscoll TR Grunstein RR Rogers NL. A systematic review of the neurobehavioural and physiological effects of shiftwork systems. *Sleep Med Rev*. 2007;11(3):179–194. 17418596 10.1016/j.smrv.2006.11.001

[48] Peršolja M Mišmaš A Jurdana M. Povezava med neprespanostjo in delazmožnostjo zaposlenih v zdravstveni negi. *Slovenian Nurs Rev*. 2018;52(1):8–17.

[49] Ruggiero JS. Correlates of fatigue in critical care nurses. *Res Nurs Health*. 2003;26(6):434–444. 14689460 10.1002/nur.10106

[50] Touitou Y Reinberg A Touitou D. Association between light at night, melatonin secretion, sleep deprivation, and the internal clock: health impacts and mechanisms of circadian disruption. *Life Sci*. 2017;173:94–106. 28214594 10.1016/j.lfs.2017.02.008

[51] Afrasiabifar A Mosavi A Mohammadian-Behbahani M Hoseinichenar N. The effectiveness of core stability exercises on nurse fatigue. *J Nurs Midwifery Sci*. 2018;5(1):9–14.

